# Triple Metachronous Malignancies with Thyroid Involvement: A Brief Overview of Five Case Reports over 20 Years of Institutional Experience

**DOI:** 10.3390/diagnostics10030168

**Published:** 2020-03-20

**Authors:** Bădan Marius-Ioan, Piciu Doina

**Affiliations:** 1 Department of Morphological Sciences, Iuliu Hațieganu University of Medicine and Pharmacy, 400012 Cluj-Napoca, Romania; doina.piciu@gmail.com; 2 Department of Endocrinology and Nuclear Medicine, “Prof. Dr. Ion Chiricuta” Institute of Oncology, 400015 Cluj-Napoca, Romania

**Keywords:** triple primary malignant tumors, thyroid carcinoma, synchronous, metachronous

## Abstract

Multiple primary malignant tumors are defined by the development of more than one malignancy in a single patient. These can be subdivided into synchronous or metachronous malignant tumors, depending on their time of appearance, relative to the first malignancy. Triple primary malignancies are a relatively rare occurrence in day-to-day practice and triple synchronous or metachronous carcinomas involving a thyroid localization are even less common. In this case series, we report our 20-year experience in diagnosing and managing five patients with triple malignant tumors with thyroid involvement.

## 1. Introduction

First described in 1889 by Billroth [1] and later extensively reported on by Warren and Gates [2] in 1932, multiple primary malignant tumors (MPMT) are defined by two or more distinct malignancies occurring in the same patient. They are further subdivided into synchronous (the second malignancy is diagnosed within the first six months of the first primary tumor) or metachronous carcinomas (the second malignancy is diagnosed after six months). Emphasis should be given to not misidentifying synchronous cancers with metastases of histologically identical cancers diagnosed at different intervals [2]. 

Approximately thirty years after the Warren and Gates publication, it was becoming clear that an advanced age allowed for more cancers to appear over time [3]. As modern-day medicine expands our lifetime and the survival of cancer patients is prolonged, these types of synchronous and metachronous malignancies should not be disregarded as mere rare peculiarities. One seemingly extensive review of the literature reports a prevalence that varies between 0.73% and 11.7% [4]. Nevertheless, studies involving more than a hundred cases of MPMT demonstrate the increasing rarity of cases with more than two malignancies [5,6,7,8]. 

The most common cancers involved in MPMT belong to the digestive system, followed by lung, head, and neck carcinomas [7]. Although often described in association with lung and breast malignancies, MPMTs involving thyroid carcinomas are among the rarest tumors described [8,9,10,11,12,13]. 

In this case report series, we briefly describe five cases of MPMT involving thyroid carcinomas as part of triple metachronous associations. 

## 2. Methods

We performed a 20-year retrospective analysis, between January 2000 and December 2019, of all cases of MPMT involving thyroid carcinomas within the “Prof. Dr. Ion Chiricuța” Institute of Oncology, Cluj-Napoca, Romania. In the last 20 years, among all 9227 cases of thyroid carcinoma from our evidence, synchronous or metachronous malignancies were present in 241 cases (2.61%) and triple association of multiple malignancies involving thyroid was present in five cases, representing 0.05%, 3 women and 2 males. The cases are presented in such a way as to not disclose any information which would lead to the identification of the person involved. The cases are described and analyzed in the following sections in decreasing order since their last admittance to our institute.

All patients signed a written consent during admission at our institute regarding their permission to process their data for research purposes.

## 3. Case Presentations

### 3.1. Case Presentation Nr. 1

**Figure 1:**
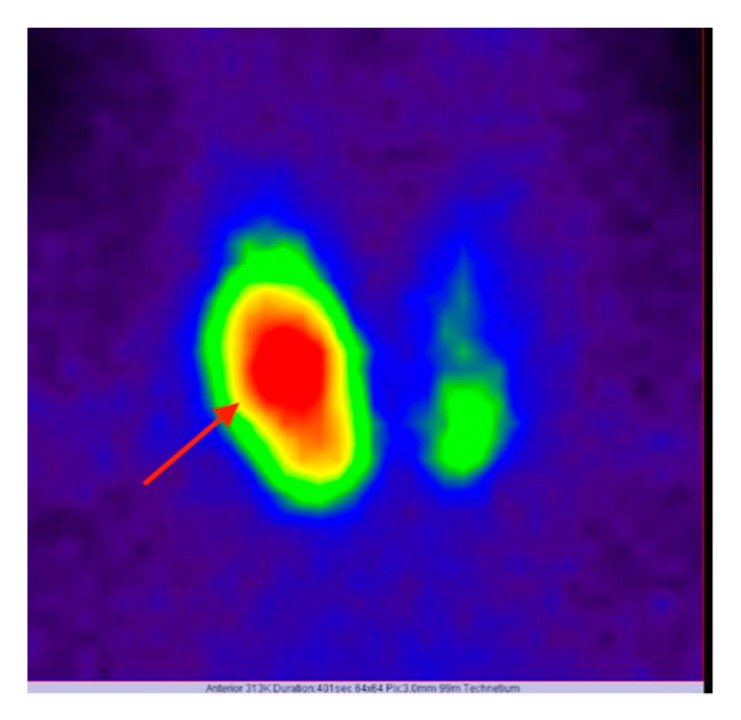
Thyroid scintigraphy with Tc-99m pertechnetate AP incidence showing a hot nodule (red arrow) in the right thyroid lobe, suggestive for thyroid adenoma (GE gamma camera LEHR); final histology exam being a hobnail variant of thyroid papillary microcarcinoma within an adenomatous nodule.

A 54-year-old Caucasian female patient with a history of high grade bilateral ovarian carcinoma Silverberg II (pT1cNxM0L1V0), with a later worsened condition (ypT3aN0MxR0V0), was investigated by computed tomography (CT) for follow-up. A thoraco-abdominopelvic CT was performed revealing a large, compressive inhomogeneous nodule in the right thyroid lobe as well as a 37 mm pulmonary nodule in the left superior lung of uncertain malignant potential, besides enlarged mediastinal lymph nodes, ascites, and carcinomatosis. Even though the marker for differentiated thyroid cancer, thyroglobulin (Tg), was not indicated for the differential diagnosis, it was determined and showed elevated levels (Tg = 260 ng/mL; N.V. = 3.5–77 ng/mL). Thyroid echography revealed a completely nodular right thyroid lobule, which was increased in size (4 cm in greatest dimension), with multiple areas of calcification, intense vascularization, and an area of central necrosis. Scintigraphy with Tc-99m pertechnetate was performed describing a hot thyroid nodule, favoring a conclusion of adenoma (Figure 1). 

**Figure 2:**
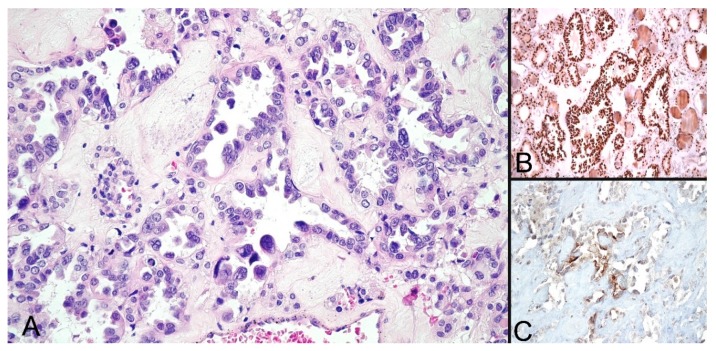
Hobnail variant of thyroid papillary carcinoma. (**A**) High power view (HPF) of hematoxylin–eosin (HE) staining showing hobnail appearance (×400). (**B**) Immunohistochemical TTF-1 stain positivity (×200). (**C**) Focal thyroglobulin immunohistochemical positivity of malignant cells (×400). Both (**B**,**C**) immunohistochemistry (IHC) markers were used to exclude other carcinomas with hobnail appearance.

The fine-needle aspiration biopsy (FNAB) was refused. The patient was scheduled for reevaluation and began chemotherapy treatment with Paclitaxel 175 mg/sqm and Carboplatin 5AUC d1, q21d for four cycles. A subsequent evaluation of the thyroid nodule revealed a toxic nodule, with a low level of TSH (0.07 mIU/L; N.V. = 04–4.5 mIU/L) and the patient started the treatment with 10 mg of Thyrozol and 10 mg of Propranolol per day. CT follow-up revealed additional lesions located at the spine (L4 and D3) with the possibility of metastases, confirmed later on by scintigraphy with 99mTc-hydroxydiphosphonate (99mTc-HDP) and single-photon emission computed tomography (SPECT-CT). Total thyroidectomy was performed and later histopathological results revealed a 3 mm hobnail variant of thyroid papillary microcarcinoma (pT1aN0) within an adenomatous nodule (Figure 2). 

Owing to a low-risk group stage I disease, according to guidelines [14], no radioiodine was indicated, and only thyroid hormone substitution was administered. After the intervention, the serum Tg level in TSH stimulated conditions (TSH 68 mIU/L) was < 0.04 ng/mL, with negative anti-Tg antibodies and negative cervical ultrasound, consistent with thyroid carcinoma complete remission. We performed a whole-body scan (WBS) with I-131 370 MBq, which was negative, confirming that the lung and bone lesions were most likely not thyroid related. In these conditions, an F18-fluorodeoxyglucose (F18-FDG) PET/CT scan was performed, revealing a 35/30 mm pulmonary lesion with a pathological F18-FDG uptake of SUVlbm Max = 4.64 and also an increased uptake in supposed bone metastases now located at L4, D12, D3, and D1 with an SUVlbm Max of 2.45 at D3. The patient underwent an endobronchial ultrasound-guided biopsy (EBUS-TBNA) and the diagnosis of ALK-negative pulmonary adenocarcinoma was revealed. Genetic studies uncovered an EGFR mutant status with c.2235_2249del deletion in exon 19, without the T790M mutation. The patient started treatment with Afatinib 40 mg 1tb/day and continued to perform CT examination with a consistent diminishing size of the lung adenocarcinoma (30 cm) and a stable number of bone metastases. The last records of the patient showed continuing regular follow-ups, being alive at the moment of this report.

We underline the particularity of this case, regarding the first diagnosis of the thyroid nodule, incidentally discovered at a routine CT follow-up, even though the nodule was large and clinically detectable. Furthermore, we would like to highlight that in the clinical arena, there is a real necessity to adapt the protocols, according to different situational aspects; in this case, the refusal of an FNAB, the thyroglobulin determination in the initial evaluation, and the use of thyroid scintigraphy. The characteristic of a “hot nodule” was confusing because a malignancy in this situation is less frequent than in a “cold nodule”. Moreover, there is a clear need to have histopathology reports whenever it is possible. Considering the awareness of two already known malignancies, the lung nodule would have been quite probably a metastasis, when in reality the third synchronous malignancy was revealed. 

### 3.2. Case Presentation Nr. 2

A 63-year-old Caucasian female patient with a history of subtotal thyroidectomy for a multinodular goiter underwent surgery for endometrial adenocarcinoma, following a worrying curettage carried out for abnormal bleeding, and received a total hysterectomy with bilateral adnexectomy. During the procedure, a 10 cm cyst, located on the left ovary, was removed. Frozen sections revealed a malignant proliferation. The final hematoxylin–eosin histopathological diagnosis was that of a synchronous endometrioid FIGO 2 carcinoma of the uterus alongside a serous papillary carcinoma arising in the left ovary. 

**Figure 3:**
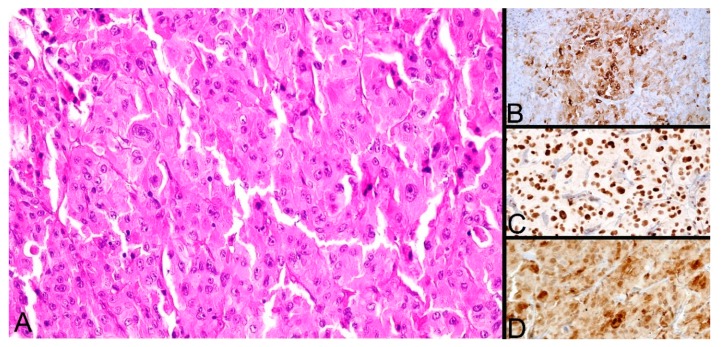
HE stain showing an oxyphilic adenoma. (**A**) Medium to large cells with abundant eosinophilic cytoplasm, characteristic for oxyphilic (Hürthle) cells, showing atypia (×200). (**B**) Focal CK19 IHC positivity (×200). (**C**) The Ki-67 (MIB-1) IHC marker for proliferation staining cell nuclei (×400). (**D**) Galectin3 cell nuclear and faint cytoplasmic positivity (×400).

Seven years later, the patient presented herself in our institute for the recurrence of the multinodular goiter. Ultrasonography demonstrated an 18.6/24.8 mm posterior, ill-defined, heterogeneous nodule with microcalcifications and Doppler vascular signal in and around the nodule. A second nodule was seen in the vicinity measuring 8/7.2 mm with a hypoechogenic halo and no Doppler signal. The patient underwent surgery for the remaining thyroid tissue and the biological material was sent for histopathological evaluation (Figure 3). The result was that of an atypical oxyphilic adenoma localized in the left remaining thyroid tissue, which also developed chronic lymphocytic thyroiditis. Subsequent immunohistochemistry (IHC) showed positivity for CK19 (Figure 3A), p53, and galectin3 (Figure 3D) on approximately 40% of the examined adenomatous surface and was negative for protein S100 and CEA with a variable Ki-67 between 6% and 18% (Figure 3C). The IHC-stained cells were thus considered malignant in nature. 

The case is suggestive because 20 years ago the indication for surgery in thyroid nodules was more frequent and easily recommended, with a less precise preoperative characterization of the malignant potential, and the indication of subtotal thyroidectomy was relatively common. Moreover, this case is representative of a very unusual and extremely rare association of a Hürthle cell (oxyphilic) variant of follicular thyroid malignancy in association with two other malignancies in the same patient. 

### 3.3. Case Presentation Nr. 3

**Figure 4:**
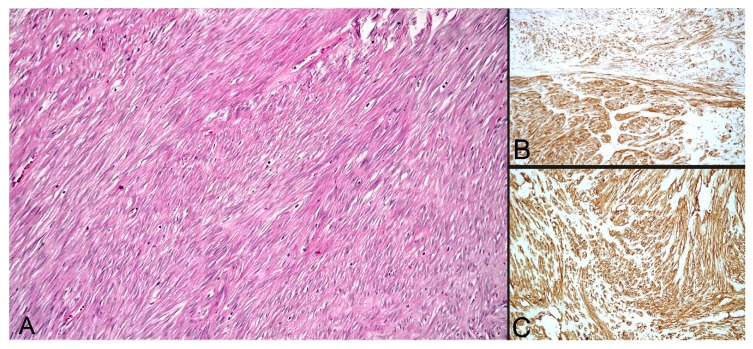
(**A**) HE stain of the gastro-intestinal stromal tumor with intertwining broad fascicles of spindle cells (×200). (**B**) IHC positivity for c-KIT (CD117) (×200). (**C**) CD34 IHC positivity (×200).

A 58-year-old Caucasian male patient with a known history of papillary thyroid carcinoma with local regional lymph node metastases was surgically treated with a total thyroidectomy; subsequently, radioiodine therapy with a total activity of 5.4 GBq was administrated. Hormone suppressive therapy and strict follow-ups, consisting of cervical ultrasounds, Tg and anti-Tg determined under stimulated conditions, as well as thoracal CTs and F18-FDG PET-CTs were done in the following years. In 2009, we performed an F18-FDG PET-CT scan revealing a 12 mm area with a moderate uptake of F18-FDG alongside a 45 mm nodular lesion, interpreted as an accessory spleen. Gastroscopy gave negative results. Later on, in 2014, the patient performed a thorax-abdominal CT scan, describing a 46 mm solid mass located within the greater curvature of the stomach next to an accessory spleen. Surgery was performed following this investigation revealing an approximately 3–4 cm polypoid tumor at the recto-sigmoid junction and a 5–6 cm subserous mass located on the posterior wall of the stomach. The following histopathological diagnosis was that of a synchronous moderately differentiated (G2) colo-rectal adenocarcinoma (pT3N2aMxL1V0, stage III B) and a gastrointestinal stromal tumor (GIST). The tumor presented as a moderately cellular mass (Figure 4) with a low mitotic index, not usually considered of high risk, but due to the initial size (45 mm) and its increases (up to 60 mm) in the relatively short period of time, the tumor was regarded as having a relatively high malignant potential. 

After the intervention, the patient was given adjuvant chemotherapy with a XELOX regimen with no adjuvant treatment recommended for the GIST. The treatment was unsuccessful in curing the disease, and in 2015, the patient went through brain surgery for metastases. The patient received external radiotherapy (with a total dose of 31 Gy/10fr) and after two more cycles of XELOX treatment developed an allergic reaction and was switched to a FOLFOX 4 regimen (a combination of oxaliplatin, folinic acid, and 5-fluorouracil). 

This case underlines the necessity of standard screenings for colorectal cancer by means of routine investigations for occult hemorrhages and colonoscopy, even if there is an already known neoplasia; we should not dismiss the probability of multiple cancers and overlook the opportunity of performing some diagnostic procedures during the protocols of follow-up, even though they are not specifically indicated in the initial diagnosis of other malignant tumors.

### 3.4. Case Presentation Nr. 4 

**Figure 5:**
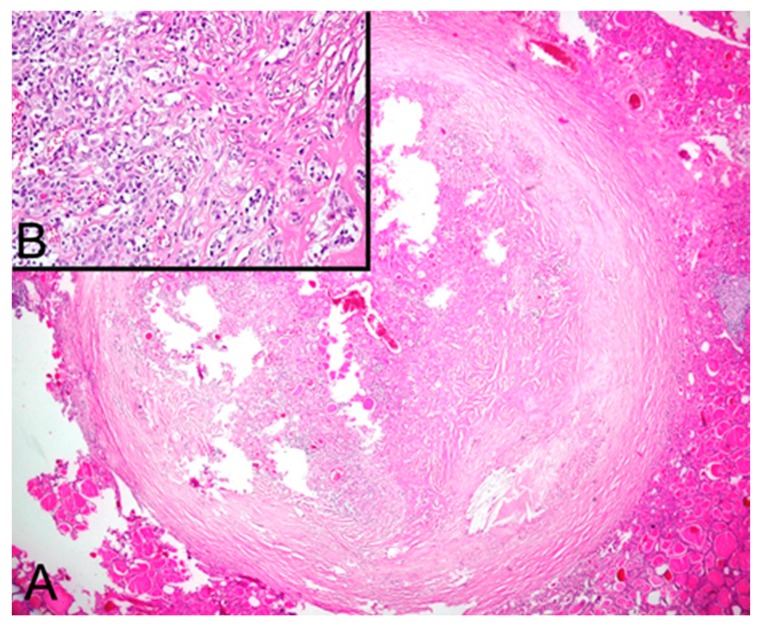
(**A**) HE stain showing a sclerosing-variant papillary thyroid carcinoma as a well-defined nodular mass (×40). (**B**) HE high power field image of tumor cells within the mass (×400).

A 52-year-old Caucasian female patient was admitted and investigated through ultrasonography for thyroid enlargement, receiving a diagnosis of multinodular goiter with a follow-up of a total thyroidectomy later on. The final histopathological evaluation of the thyroid tissue revealed a papillary thyroid microcarcinoma (Figure 5) alongside the confirmation of the clinical diagnosis.

The patient had a history of a recurrent vulvar keratinizing squamous cell carcinoma that was diagnosed and first operated nine years earlier (vulvectomy with right inguinal lymphadenectomy). Later on, after 7 years, the patient developed a uterine endometrioid carcinoma (FIGO 3, pT2a), which was surgically removed through a total hysterectomy with bilateral adnexectomy, and received adjuvant external radiotherapy (with a total dose of 50 Gy). The patient underwent surgery, outside of the country, for a radical bilateral vulvectomy in 2010. Afterward, the same year, the patient was admitted into our institute for local relapse of the vulvar carcinoma. The following year, a CT scan was performed and a disease-free status was confirmed. The patient was last being treated for post-therapeutic myxedema, type 2 diabetes controlled through diet management, and second-degree hypertension.

This case underlines the necessity for whole-body evaluations in patients with a long history of malignancy in one area of the body. In such patients, we must not concentrate on the disease and its local reoccurrences and try to maintain a more holistic and open-minded approach to patient care. The thyroid microcarcinoma in our case was a serendipitous discovery.

### 3.5. Case Presentation Nr. 5 

A 46-year-old Caucasian male patient was admitted in 2005 and surgically treated for a papillary thyroid carcinoma with a total thyroidectomy and selective lymphadenectomy (pT3N1aMx), followed by 3.7GBq I-131 and hormone suppressive therapy. 

**Figure 6:**
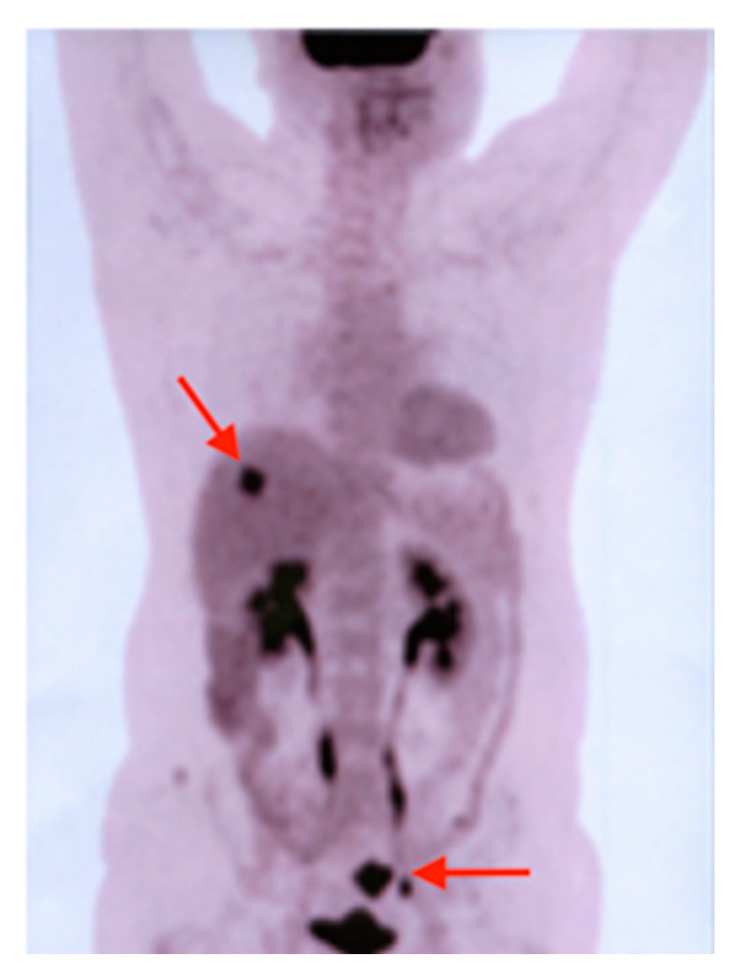
MIP image of F18-FDG PET-CT revealing a lesion in the sigmoid colon (lower-right red arrow) and a lesion of 21 mm in the VIIth liver segment (upper-left red arrow), consisting of a colon malignant tumor with liver metastases.

**Figure 7:**
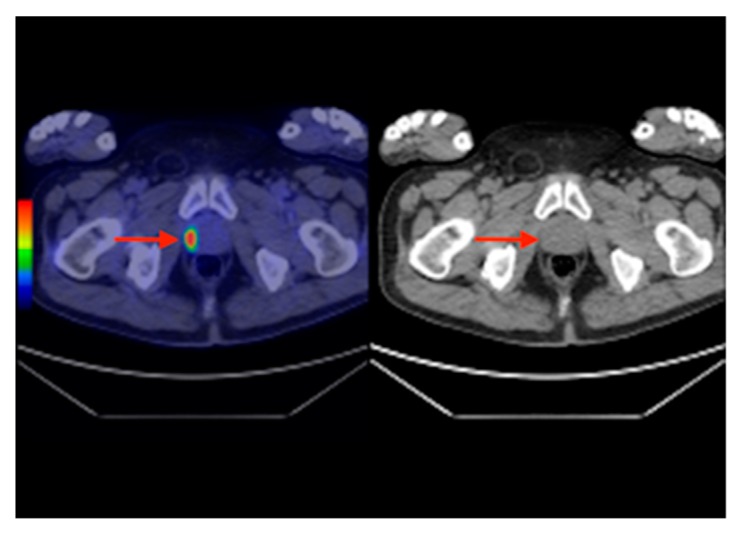
Axial section F18-FDG PET-CT (left) and CT (right) revealing an increased uptake in the right prostate lobe (red arrows) suggesting a primary prostate carcinoma, confirmed in histology.

In 2007, an F18-FDG PET-CT scan was performed, which revealed a 30/28 mm focal increase of uptake in the sigmoid colon and a lesion of 21 mm in the VIIth liver segment, consisting of a colon malignant tumor with liver metastases (Figure 6). The patient was submitted for hemicolectomy and liver metastases resection, followed by chemotherapy. In 2013, during the routine examination, we found a focal uptake in the right prostatic lobe. After the prostatectomy, the histopathological result showed a prostate adenocarcinoma, occurring in a patient with already known thyroid and colon carcinomas (Figure 7). 

This case underlines the important contribution of the F18-FDG PET-CT scan during the follow-up of malignant tumors. 

### 3.6. Overview

A brief overview of the selected cases can be seen in the following table (Table 1):

## 4. Discussions

Our study aimed to describe triple MPMTs with thyroid involvement as a single-center experience. Other institute-level screenings have been conducted regarding MPMTs with double and triple cancer associations but they were not concentrated on triple thyroid-related malignancies [9,15,16]. Moreover, several case reports can be found in the scientific literature describing associations between thyroid carcinomas and at least two other malignancies in the same patients [17,18,19]. Somewhat unusual associations have been described between thyroid carcinomas and renal cell carcinoma or heart leiomyosarcomas [20,21]. Our search for relevant articles concerning triple cancer associations with thyroid involvement showed mostly single case reports and can be summarized by year of appearance and location of the second and third cancer in Table 2.

Our approach to describe these cases has been more focused on the medical imaging side of the diagnosis with a histopathology confirmation, rather than on biological markers and clinical symptoms. The importance of PET-CT imaging should be underlined as two of our cases had additional cancers discovered by routine PET–CT screenings for their initial diagnostic. This approach is more in line with current trends of relying more on imaging data [27] complemented with pathology reports. One such example is the case of combining different imaging techniques with dual radiopharmaceuticals in multiple carcinoma diagnostics for efficient evaluation [37]. More evidence in this regard is provided by Ishimori et al. in a study concerning 1912 patients who underwent whole-body PET-CT scans. A high F18-FDG uptake, suggestive for new, undiagnosed malignancies, was found in 79 (4.1%) cases. Thyroid histopathological proven malignancies were found in six cases (second most common after lung) [38]. Unexpected F18-FDG uptake has been previously described particularly for lesions regarding the gastro-intestinal tract, leading to the discovery of malignant or premalignant proliferations [39]. Lastly, the importance of PET-CT hybrid imaging in diagnosing MPMTs is shown by Even-Sapir et al. who described 44 new malignancies found in 115 patients who underwent PET-CT evaluation for suspected second primary malignancies [40]. Following these recent publications, coupled with our own findings, it seems reasonable to consider that follow-up PET-CT screenings play a major role in the diagnostics of more than one primary malignancy in known cancer patients. Serum biomarkers are also potentially helpful and non-irradiating but lack the organ-specificity of hybrid imaging techniques. Several cancer biomarkers present elevated levels in more than one type of cancer, suggestive rather than diagnostic for a second primary malignancy, ultimately leading to further investigations through imaging techniques. 

**Figure 8:**
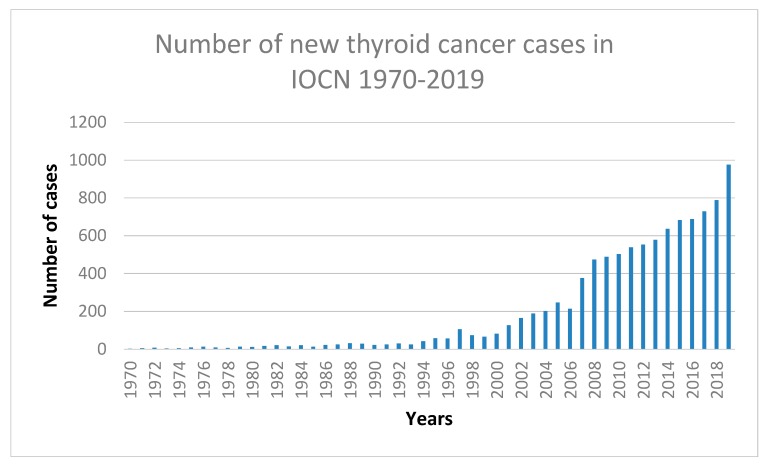
Graphical representation of thyroid cancer cases between 1970 and 2019 in the “Prof. Dr. Ion Chiricuța” Institute of Oncology (IOCN), Cluj-Napoca, Romania.

Even though our institute’s data show an increase in new cases of thyroid cancer each year (Figure 8), with 9227 total cases of thyroid malignancies in the past 20 years, triple multiple primary malignancies, one being a thyroid cancer, were very rare (0.05%). Double MPMTs with thyroid involvement were more frequent with 241 reported cases (2.61%). 

With the exception of two cases, most of our patients were female with a median age of 53 at the time of the thyroid carcinoma diagnosis. This could explain why the most common associations of thyroid localized carcinomas were, in our case, represented by gynecological malignancies (two cases with endometrioid endometrial carcinoma and two with serous papillary ovarian carcinoma, one vulvar squamous carcinoma, and one cervical adenocarcinoma). Even though Piciu et al. [14] showed an increased relative risk of developing a breast, uterine, or ovarian cancer after a primary differentiated thyroid carcinoma, in our case series, thyroid carcinomas developed after at least one gynecological carcinoma in all female patients. If we are looking for two malignancies, the most frequent association of MPMT in our institute was thyroid carcinoma and breast cancer, but in the association of triple cancers, this commune presentation was completely missing. Despite the fact that in larger populations the sex distribution seems to be more evenly spread [9], a female predominance with a similar age range has been previously reported and could be one of the reasons why papillary thyroid carcinomas seem to be more likely associated with breast malignancies [41]. Even though none of our cases featured breast malignancies, this association has been debated in the past since the 1960s [42]. Another possible explanation for this association could be that thyroid carcinomas are more prevalent in women [43,44]. Corso et al. [11] described a higher risk of developing a secondary thyroid malignancy in breast cancer patients who underwent hormonal therapy. An explanation could be the estrogen receptor that seems to be involved in both of the mechanisms that drive their appearance [12]. Attempts to characterize breast and thyroid tumors in order of emergence have not led to conclusive results, with an equal distribution in eight cases, but the widespread association of these malignancies suggests the importance of establishing a screening program for patients suffering from either of the primary diseases [13]. 

In our case series, except for Case Nr. 2, triple metachronous cancer associations seem to develop in a relatively short period of time from one to the other (Table 1). No obvious pattern of the disease-free interval was observed from our study samples. The most common histological type of thyroid malignancy was that of papillary thyroid carcinoma. An ample retrospective study performed in our institute showed a good 10-year survival (80.4%) of patients diagnosed and treated with this type of pathology [44]. Two of our cases presented with synchronous malignancies, none of which were thyroid carcinoma associations. Although not thyroid-related, similar findings have been also reported by Kapoor et al. [45], with two months and three years of disease-free intervals. In some studies, nonetheless, more metachronous MPMTs were to be expected as they have been previously reported to be more common than synchronous tumors [9,16]. 

Although the patient reviewed in our first case never received a biopsy confirmation of the bone metastases, they were most likely of pulmonary origin considering the extension and severity of the presentation. Nevertheless, in our experience, as well as in best practice, it is never a wrong decision to consider even the most remote of possibilities when dealing with differential diagnoses for bone metastases in a former thyroid carcinoma patient [46]. 

It would be noteworthy to study different types of common oncoprotein expressions in these carcinomas to see if there is any chance of syndrome-like associations. With regards to our fourth case presentation, Shibutani et al. [30] described a similar case of malignancy association and found both of the proteins expressed in the thyroid as well as in the rectal cancer tissue after performing IHC studies for p21 and p53. The p21 protein also stained surrounding adenomatous regions in the colon as well. Their variant of gastric malignancy (poorly differentiated adenocarcinoma) did not express the oncoproteins. In our case, a GIST would also be unlikely to express such proteins as shown by Ihle et al. [47]. Of course, others have also noted the association of thyroid and gastric carcinomas, as digestive MPMTs, alongside pulmonary neoplasms, have been reported to be more frequent [7], although contradicting data exists on this matter and seems to rely heavily on the study population [15]. Genetical studies in synchronous lung and thyroid carcinomas, in a triple MMPT case, showed that both of them carried EGFR and BRAF mutations but with distinct mutation profiles [23]. 

These types of association may not only have an important role in describing potential common pathways in pathogenesis but also, and more importantly, could be a way of applying a lesser number of targeted therapies in the future, as no definitive protocol currently exists on how to manage MPMTs from this point of view [48]. 

This study suffered from several identifiable limitations, some of which include the inadequate organization of the patient files, limited clinical data on symptoms and missing serum biomarkers in some cases as they were performed in tertiary private hospitals or institutions during long periods. Genetic or IHC studies for oncoproteins were not extensively utilized in the majority of our patients, consequently making in-depth analyses of the cases difficult to achieve. New 2017 World Health Organization modifications on papillary thyroid carcinomas (PTC) variants were taken into account. Although our study did not have follicular variants of papillary thyroid carcinomas, retrospective studies similar to ours could be influenced by the 2017 WHO modifications, as it has been shown to downgrade formerly PTC classified tumors by as much as 31% [49].

In conclusion, MPMTs should not be neglected in patients treated for a primary malignancy, even in the early years after treatment. Even within our limited sample size, we can observe that MPMTs can be detected through routine investigations (mostly through imaging techniques) even before early symptoms of the second malignancy. When dealing with female cancer patients, especially with breast cancer survivors or women diagnosed with gynecological carcinomas, care should be taken to screen for possible occult thyroid malignancies. 

## Figures and Tables

**Table 1:** Summary of the cases.

Case No.	Location of Malignancy	Histology	Gender	Age at Thyroid Carcinoma Diagnosis	Initial Diagnosis (Year)	Disease-Free Interval (Years)
Case 1	ovarian	serous adenocarcinoma	female	53 years	2017	NA
	thyroid	papillary microcarcinoma			2018	1
	lung	acinar adenocarcinoma			2019	1
Case 2	endometrial	endometrioid carcinoma	female	71 years	2005	NA
	ovarian	serous adenocarcinoma			2005	0
	thyroid	Hürthle cell carcinoma			2019	14
Case 3	thyroid	papillary carcinoma	male	58 years	2008	NA
	stomach	gastro-intestinal stromal tumor			2014	6
	colon	adenocarcinoma			2014	0
Case 4	vulva	keratinizing squamous cell carcinoma	female	53 years	1993	NA
	endometrial	endometrioid carcinoma			2000	7
	thyroid	papillary carcinoma			2002	2
Case 5	thyroid	papillary carcinoma	male	46 years	2005	NA
	colon	adenocarcinoma			2007	2
	prostate	adenocarcinoma			2013	6

*NA = not applicable; no prior known malignancy.

**Table 2:** Summary of Pubmed indexed articles of triple malignancies with thyroid involvement.

Investigators	Year of Publication	Location of Second and Third Malignancies besides the Thyroid Carcinoma
Peng et al. [22]	2019	Colon; Kidney
Peng et al. [23]	2018	Lung; Kidney
Kikuchi et al. [24]	2017	Stomach; Breast
Singh et al. [25]	2016	Larynx; Lymph nodes
Cohen et al. [26]	2016	Kidney; Skin
Oh et al. [27]	2015	Stomach; Kidney
Adams and Caffrey [28]	2014	Larynx; Thyroid (second)
Lee et al. [17]	2010	Rectum; Uterus
Omür et al. [29]	2008	Breast; Stomach
Iqbal et al. [18]	2008	Larynx; Lung
Rai et al. [21]	2007	Kidney; Prostate
Hamada et al. [19]	2000	Lung; Stomach
Fukuoka et al. [20]	2000	Heart; Lung
Shibutani et al. [30]	1994	Stomach; Rectum
Arimura et al. [31]	1989	Uterus; Lung
Ohi et al. [32]	1988	Skin; Brain
Watanabe et al. [33]	1984	not specified
Yamamoto et al. [34]	1983	Ovary; Brain
Nemoto et al. [35]	1977	Kidney; Nasopharynx
Ishii et al. [36]	1977	Stomach; Stomach
